# Solid-phase microextraction of endogenous metabolites from intact tissue validated using a Biocrates standard reference method kit

**DOI:** 10.1016/j.jpha.2022.09.002

**Published:** 2022-10-08

**Authors:** Runshan Will Jiang, Karol Jaroch, Janusz Pawliszyn

**Affiliations:** a Department of Chemistry, University of Waterloo, Waterloo, N2L 3G1, Canada; b Department of Pharmacodynamics and Molecular Pharmacology, Faculty of Pharmacy, Collegium Medicum in Bydgoszcz, Nicolaus Copernicus University in Toruń, Bydgoszcz, 85-089, Poland

**Keywords:** Solid-phase microextraction, Solvent extraction, Metabolomics, Sample preparation, In vivo sampling

## Abstract

Improved analytical methods for the metabolomic profiling of tissue samples are constantly needed. Currently, conventional sample preparation methods often involve tissue biopsy and/or homogenization, which disrupts the endogenous metabolome. In this study, solid-phase microextraction (SPME) fibers were used to monitor changes in endogenous compounds in homogenized and intact ovine lung tissue. Following SPME, a Biocrates AbsoluteIDQ assay was applied to make a downstream targeted metabolomics analysis and confirm the advantages of in vivo SPME metabolomics. The AbsoluteIDQ kit enabled the targeted analysis of over 100 metabolites via solid-liquid extraction and SPME. Statistical analysis revealed significant differences between conventional liquid extractions from homogenized tissue and SPME results for both homogenized and intact tissue samples. In addition, principal component analysis revealed separated clustering among all the three sample groups, indicating changes in the metabolome due to tissue homogenization and the chosen sample preparation method. Furthermore, clear differences in free metabolites were observed when extractions were performed on the intact and homogenized tissue using identical SPME procedures. Specifically, a direct comparison showed that 47 statistically distinct metabolites were detected between the homogenized and intact lung tissue samples (*P* < 0.05) using mixed-mode SPME fibers. These changes were probably due to the disruptive homogenization of the tissue. This study's findings highlight both the importance of sample preparation in tissue-based metabolomics studies and SPME's unique ability to perform minimally invasive extractions without tissue biopsy or homogenization while providing broad metabolite coverage.

## Highlights

• A Proof-of-concept study demonstrating SPME coupled to a Biocrates targeted metabolomics kit is presented for the first time.
• SPME coupled to the Biocrates bioassay reveals drastic metabolome changes between intact and homogenized tissues.
• SPME offers coverage of a great variety of analytes.
• SPME is a desirable approach for delicate in vivo metabolomics studies.

## Graphical abstract

**Figure:**
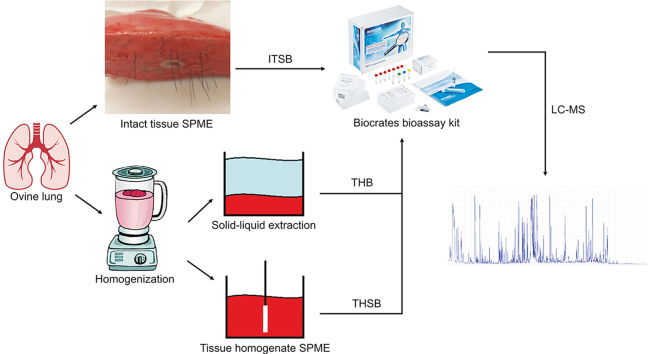

## 1 Introduction

Metabolomics is the systematic study of low-molecular-weight molecules in a biological system. Typically, the primary aim of metabolomics is to quantify the effects of physiological and biochemical changes on cellular metabolism. For example, metabolomics analysis can be used to assess the survival outcomes of donor lungs in transplant patients [1]. As metabolomics gains more interest in the fields of biology and biomedicine, researchers must contend with new challenges and considerations related to sample acquisition, storage, and preparation. For instance, Roszkowska et al. [2] observed differences in the metabolomic coverage provided by ex vivo solid-phase microextraction (SPME) and solid-liquid extraction (SLE) when the two methods were applied to analyze fish tissue. Specifically, they found that SLE resulted in the detection of more lipids due to the release of compounds from protein complexes and the disruption of the bilayer membrane. A similar study showed that metabolomics using in vivo SPME enabled the extraction of unstable fish tissue metabolites that could not be detected by traditional methods. This advantage was enhanced by immediately following up in vivo SPME with instrumental analysis to avoid changes in the sample induced during storage [3]. Even with simpler sample matrices, such as blood, urine, and other biological fluids, numerous preanalytical variables have been shown to result in biases that can adversely impact the outcome of qualitative and quantitative analyses [4]. As such, direct sampling and sample preparation techniques are highly desirable.

When applied in vivo, SPME is a powerful and minimally invasive analytical technique that allows chemical biopsies to be performed directly on a living system. In vivo SPME typically utilizes a miniature fiber geometry, which can be inserted into the target tissue to obtain metabolomic information in its current physiological state without alteration [5]. In addition, SPME's metabolism-quenching effect prolongs the stability of bioactive metabolites which can be stored on-fiber, transported, and frozen until analysis [5,6]. SPME is also compatible with several different extraction phases, offering a broad range of analyte coverage [7], while its nonexhaustive nature helps to minimize changes in the studied biological system [6]. The latter feature is key in differentiating SPME from exhaustive extraction methods such as liquid-liquid extraction and solid-phase extraction, which typically involve destructive sampling of the studied system. For instance, Napylov et al. [8] successfully extracted up to 52 oxylipins from the brain tissue of live and conscious rats via SPME, which was the largest panel of oxylipins detected in vivo to date. The success of their study was largely due to SPME's ability to overcome the chemical limitations of the membranes used in conventional in vivo microdialysis. Furthermore, Napylov et al. [8] noted that over 24 of the oxylipins had been exclusively detected postmortem, with only three detected in living rats via in vivo SPME. This observation, combined with the success of Napylov et al.’s study, highlights the significant advantages of in vivo SPME compared to conventional destructive sampling methods. Further applications of in vivo SPME in animal models yielded interesting findings, such as significantly different endogenous metabolomes in the brains, livers, kidneys, and muscles of different strains of lab mice [9]. In another study, in vivo SPME was successfully utilized to measure and correlate the local concentrations of the cognitive enhancement drug, donepezil, and its target enhancement metabolite, acetylcholine, for the first time in a live primate (*Macaca mulatta*) [10]. Following years of exploratory work assessing the application of in vivo SPME in animal models, researchers shifted their attention to explore human clinical applications of this technology in the last decade. In 2021, Bogusiewicz et al. [11] demonstrated, for the first time, the viability of applying SPME to sample brain tissue in a conscious human patient. They successfully performed chemical biopsies in vivo by inserting four SPME fibers into the patients' brain tissue prior to stereotactic biopsy. This approach enabled them to avoid causing any extra damage, while still allowing the standard procedure to be performed in parallel during the operation. The obtained lipidomic data were characteristic of brain tissue, thus confirming SPME's potential as a complementary tool for collecting information in vivo. Similarly, in vivo SPME has been used for therapeutic drug monitoring (TDM) and metabolomics in the lungs. To this end, SPME fibers coated with a biocompatible C8-sulfonate (mixed-mode) extraction phase were used to quantify concentrations of the chemotherapeutic agent, doxorubicin, in the lungs undergoing perfusion during localized chemotherapy [12,13]. The desorption solutions obtained from the fibers used in these analyses were then subjected to further untargeted metabolomics analysis via ultra-high-performance liquid chromatography coupled with high-resolution mass spectrometry [12].

SPME enables quantification through kinetic calibration in in vivo experiments, but this ability depends on certain requirements. Typically, internal standards are preloaded onto the fiber and the quantity of target analytes is calculated based on the desorption and adsorption rates of the internal standard and analyte, respectively. To ensure isotropy in the analytes’ adsorption and desorption to and from the SPME fiber, the selected internal standards must share similar physicochemical properties with the target analytes; thus, deuterated internal standards of the target analytes are typically the optimal choice. As a result, kinetic calibration is a suitable method for applications requiring the quantification of a limited number of compounds, such as TDM or pharmacokinetic studies in blood [14,15]. However, since metabolomic profiling deals with an enormous number of compounds in various biological matrices, kinetic quantification using internal standards is often impractical. Additionally, even if analytically suitable standards could be chosen, utilizing internal standards in vivo is challenging for safety reason. Instead, matrix-matched calibration is often an appropriate alternative. Roszkowska et al. [16] demonstrated that comparable analyte responses can be achieved for various chemotherapeutic agents when using lamb tissue homogenate to simulate in vivo conditions. Thus, in vivo SPME can be used as a quantitative analytical technique when coupled with in vitro matrix-matched calibration.

Nonetheless, some challenges remain in untargeted metabolomics, with analytical variation ranking among the most significant. Instrumental signal variations can occur due to numerous factors, such as inherent imperfections in the analytical instruments and varying ion suppression caused by drastic differences in analyte composition between individual samples. This difference is further exaggerated when handling a large cohort, or inter-batch, inter-day, or inter-laboratory variability [17,18]. In addition, metabolite annotation during data mining is a major bottleneck in untargeted metabolomics. To alleviate this bottleneck, commercial metabolomic assays capable of quantifying hundreds of key metabolites from various compound classes can be used [18]. In this study, an Absolute p180 kit (Biocrates, Innsbruck, Austria) was used for the quantitative analysis of nearly 200 endogenous compounds, including amino acids, biogenic amines, monosaccharides, acylcarnitines, glycerophospholipids, and sphingolipids. The kit contains a 96-well plate, which is used to introduce internal standards to the unknown samples, as well as calibration standards and three levels of quality control (QC) samples. In this study, we used a Vanquish UHPLC coupled with a Vantage TSQ mass spectrometer (Thermo Fisher Scientific Inc., Waltham, MA, USA) for both the liquid chromatography-mass spectrometry (LC-MS) and flow injection analysis (FIA) portions of the assay. The LC-MS portion aimed to detect amino acids and biogenic amines, whereas the FIA portion was intended to detect monosaccharides, acylcarnitines, glycerophospholipids, and sphingolipids. Currently, all assays sold by Biocrates are based on liquid extractions, which require homogenization for tissue analysis. As discussed earlier, this process invariably results in changes to the metabolome.

This proof-of-concept study has two primary objectives: to show that SPME can be combined with a Biocrates AbsoluteIDQ kit to detect various analytes in targeted tissue metabolomics and to demonstrate the advantages of intact tissue sampling compared to homogenized tissue sampling.

## 2 Materials and methods

### 2.1 Chemicals and reagents

An AbsoluteIDQ p180 kit was purchased from Biocrates Life Sciences (Innsbruck, Austria). Further details related to the contents of the kit can be found at https://biocrates.com/absoluteidq-p180-kit/. Pro analysis (P.A.)-grade phosphate buffered saline (PBS) tablets, LC-MS-grade ethanol, methanol, acetonitrile, and 2-propanol were purchased from Thermo Fisher Scientific Inc. (Waltham, MA, USA). P.A.-grade phenylisothiocyanate (PITC), pyridine, LC-MS-grade ammonium acetate (MilliporeSigma, Burlington, MA, USA), and formic acid were purchased from Sigma-Aldrich (St. Louis, MO, USA). Ovine lungs were chosen as the target organ tissue and were purchased fresh from a local butcher shop.

### 2.2 LC, FIA, and tandem MS

Biogenic amines and amino acids were analyzed via Thermo Vanquish Horizon LC coupled with a Thermo Vantage TSQ MS system (Waltham, MA, USA). The LC-MS system was managed using Thermo Xcalibur 4.0, Chromeleon 7.2.6, and Thermo TSQ Series 2.6 Tune Master instrument control software. The chromatography method was adopted from the AbsoluteIDQ p180kit (Biocrates Life Sciences, Innsbruck, Austria) with the following modification: the chromatographic separation process was optimized and performed on a C_18_ reverse-phase high performance liquid chromatography (HPLC) column (100 mm × 2.1 mm, 3 μm particles) at 50 ± 0.5 °C and a C_18_ reverse-phase HPLC guard column (10 mm × 2.1 mm, 3 μm particles) (both purchased from Biocrates Life Sciences, Innsbruck, Austria). Mobile phase A comprised of ultrapure water and 0.2% (*V/V*) formic acid, and mobile phase B comprised of acetonitrile and 0.2% (*V/V*) formic acid. Separation was achieved using the following multistep gradient: starting at 0% B until 0.25 min, followed by a linear increase to 12.0% B until 1.5 min, then a linear increase to 17.5% B until 2.7 min. A steep linear increase to 50.0% B took place until 4 min, followed by a steeper increase to 95% B until 4.5 min. B was then maintained at 95% until 5.25 min, followed by a sharp linear decrease back to 0.0% B until 5.4 min. Last, the method ended with a 2.6 min equilibration stage at 0.0% B (8 min total). A flow rate of 0.8 mL/min was maintained throughout the gradient for each 5.0 μL injection. All samples were kept at 4 °C in the LC autosampler prior to injection. The autosampler syringe was washed with acetonitrile, methanol, isopropanol, and water (50:20:15:15, *V*/*V*/*V*/*V*) for 5.0 s at 32.0 μL/s before and after each injection.

Acylcarnitines, glycerophospholipids, sphingolipids, and hexoses were analyzed via FIA on the same LC-MS system in both positive and negative modes according to the kit's recommendation. All mobile phases used in the LC-MS and FIA (FIA additive provided in the kit) analyses were degassed via ultrasonication for 15 min in a VWR Scientific Aquasonic 75HT Ultrasonic Cleaner (West Chester, PA, USA).

### 2.3 Sample preparation

Three sample preparation and extraction methods were tested in this study: 1) tissue homogenization followed by SLE coupled with the Biocrates kit (THB); 2) tissue homogenization followed by SPME coupled with the Biocrates kit (THSB); and 3) intact tissue extraction via SPME coupled with the Biocrates kit (ITSB). In methods 2) and 3), the Biocrates AbsoluteIDQ p180 kit was used for metabolite detection, quantification, and QC for SPME.

Chilled ovine lung tissue samples (4 °C) were diced up to remove the major bronchi and adipose tissue. A uniform suspension was obtained using a generic tabletop blender to homogenize approximately 200 g of the tissue with small amounts of dry ice to avoid heating. The homogenate was then mixed with 0.01 M PBS at a ratio of 1:3 (mg tissue/μL solvent) on a benchtop vortexer to produce the final homogenate [19]. PBS was chosen as the extraction phase for SLE because organic solvents are generally incompatible with SPME, and PBS offers the added advantage of the favorable extraction of more polar analytes, such as amino acids, biogenic amines, and short-chain lipids compared to organic extraction phases. The supernatant for THB analysis was collected by centrifuging a portion (∼10 mL) of each tissue homogenate sample at 10,000 *g* for 5 min using a swing-bucket rotor inside an Eppendorf Centrifuge 5804R (Hamburg, Germany) (see below).

### 2.4 SLE- and SPME-AbsoluteIDQ p180 kit protocol

The in vivo SPME methodology used in this study was adopted from Bojko et al. [12] with minor changes. Specifically, the present study was conducted using nitinol-based SPME fibers coated with a C8-sulfonate strong cation exchange (SCX) mixed-mode extraction phase (Supelco, Bellefonte, PA, USA). The coated wires were cut into ∼4 cm fibers and stripped such that the coating length extended 15 mm from the tip. The fibers were then cleaned with a solution comprised of acetonitrile, methanol, isopropanol, and water (30:30:30:10, *V/V/V/V*) for 30 min on a BenchMixer Multi-Tube Vortexer (Benchmark Scientific, Edison, NJ, USA) at 1,500 rpm. After cleaning, the fibers were placed in a methanol:water (50:50, *V/V*) preconditioning solution for 15 min at 1,500 rpm, and then stored in a fresh preconditioning solution until use. Four fibers were briefly rinsed with ultrapure water (∼5 s) to remove any surface solvent and then inserted into the tissue homogenate prepared in Section 2.3 (THSB). The same rinsing process was applied to an additional four fibers, which were then inserted into intact ovine lungs (ITSB) for 20 min at ∼18 °C (instrument room temperature) for static extraction. Following extraction, the fibers were removed from the sample, rinsed in ultrapure water for 5 s to remove any particulates and macromolecules that may have attached to the surface of the coating via nonspecific binding, and briefly dried with a Kim wipe. Next, the fibers were desorbed in an 80-μL acetonitrile and water (80:20, *V/V*) solution for 60 min at 1,500 rpm. PBS blanks, 10-μL aliquots of the solvent extraction for THB, and 40 μL of desorption solution from THSB and ITSB were added to the kit plate according to the instructions provided in the user manual. Similarly, the PITC derivatization of amino acids was conducted according to the instructions provided in the user manual. Four replicates were performed for each sample type (*n* = 4).

### 2.5 Data processing and statistical analysis

The raw instrumental data from the LC-MS and FIA analyses were processed using MetIDQ, Version Boron (Biocrates Life Sciences, Innsbruck, Austria), following the directions in Sections 5 and 6 of the user manual (page 180). Due to the changes made to the default kit protocol to accommodate the integration of SPME as an upstream sample preparation and extraction step, the processed raw data (i.e., signal intensity for FIA, peak area, and area:internal standard ratios for LC-MS) from MetIDQ were subjected to further manual sorting in Excel (Microsoft Office 365, Albuquerque, NM, USA). During this sorting step, compounds were excluded if the internal standard-corrected data (response of analyte/response of internal standard) had a signal-to-noise ratio of less than 3 or a relative standard deviation (RSD%) exceeding 30%. The sorted LC-MS and FIA data were then combined and uploaded for data preprocessing on Metaboanalyst 5.0. Pareto scaling and log transformation was employed to account for large values for certain metabolites. Principal component analysis (PCA) was also performed using Metaboanalyst 5.0. Data from all three groups (ITSB, THSB, and THB) were subjected to nonparametric one-way analysis of variance (Kruskal-Wallis test) with *P* < 0.05 on Statistica, version 13.3. (TIBCO Software Inc., Palo Alto, CA, USA).

## 3 Results and discussion

**Fig. 1:**
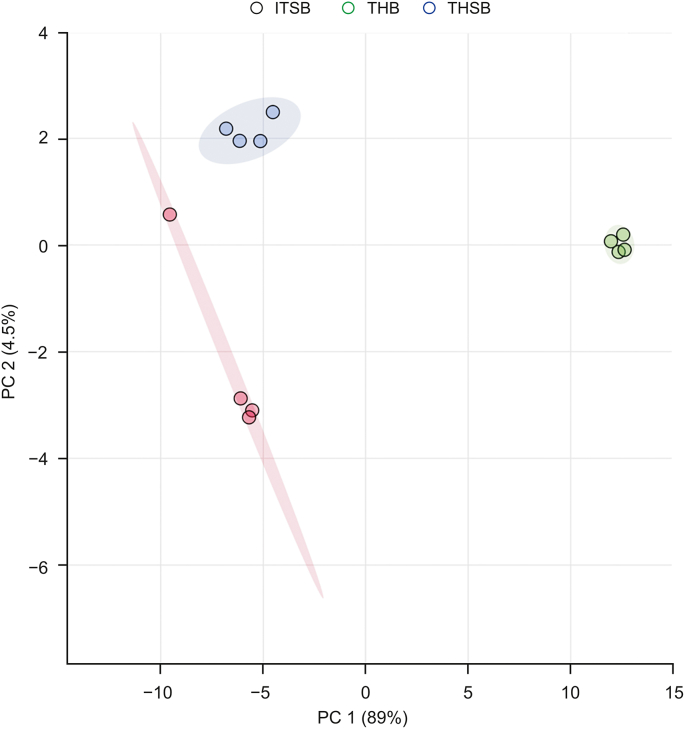
Principal component analysis of THB, THSB, and ITSB sample preparation techniques. THB: tissue homogenization followed by solid-liquid extraction (SLE) coupled to the Biocrates kit; THSB: tissue homogenization followed by solid-phase microextraction (SPME) coupled to the Biocrates kit; ITSB: intact tissue extraction via SPME coupled to the Biocrates kit.

The results of a global PCA revealed distinct separations between the three sample preparation methods (Fig. 1). As expected, the biggest separation (along principal component 1 axis) was observed for the extractions from homogenized lamb lung tissue via conventional SLE (THB) and the two extractions using SPME fibers (THSB and ITSB). This difference was mostly due to the considerably larger quantity of analytes extracted by the exhaustive SLE method compared to the nonexhaustive SPME technique. A complete table of compounds and their relative abundances can be found in Table S1. Exemplary LC-MS and FIA chromatograms for all three method groups can be found in Figs. S1−S9.

**Fig. 2:**
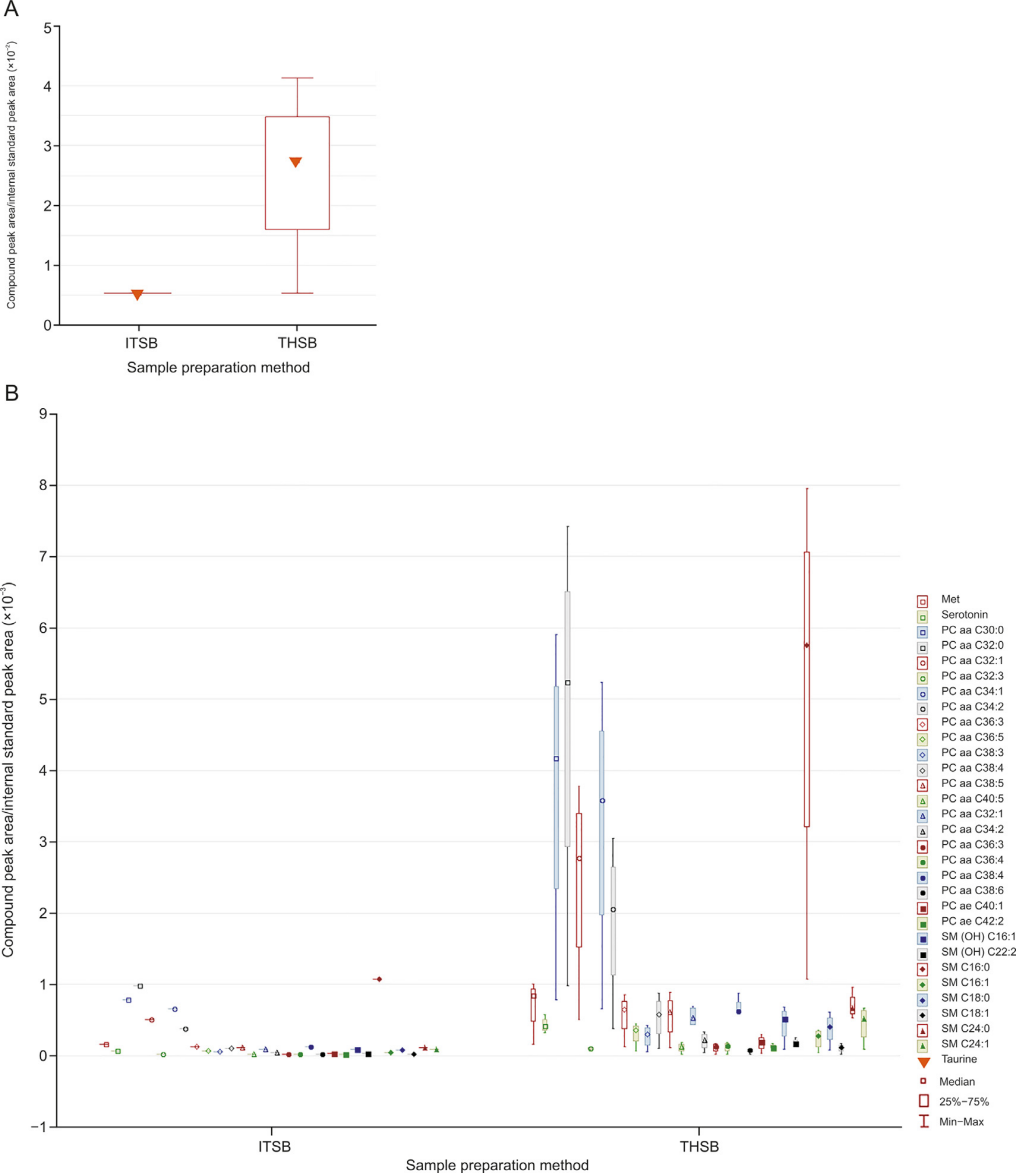
Compounds detected in greater abundance from homogenized tissue (THSB) than intact tissue (ITSB) by solid-phase microextraction (SPME) coupled to the Biocrates kit. The y-axis in (A) is over an order of magnitude larger than that in (B) and therefore shown separately. Y-axis values were preprocessed with Metaboanalyst 5.0 online software. The legend lists the compounds in a descending order, corresponding to their appearance on the graph from left to right. THSB: tissue homogenization followed by SPME coupled with the Biocrates kit; ITSB: intact tissue extraction via SPME coupled with the Biocrates kit; Met: methionine; PC: phosphatidylcholine; PC aa: diacyl-PC; PC ae: acyl-alkyl-PC; SM: sphingomyelin.

**Fig. 3:**
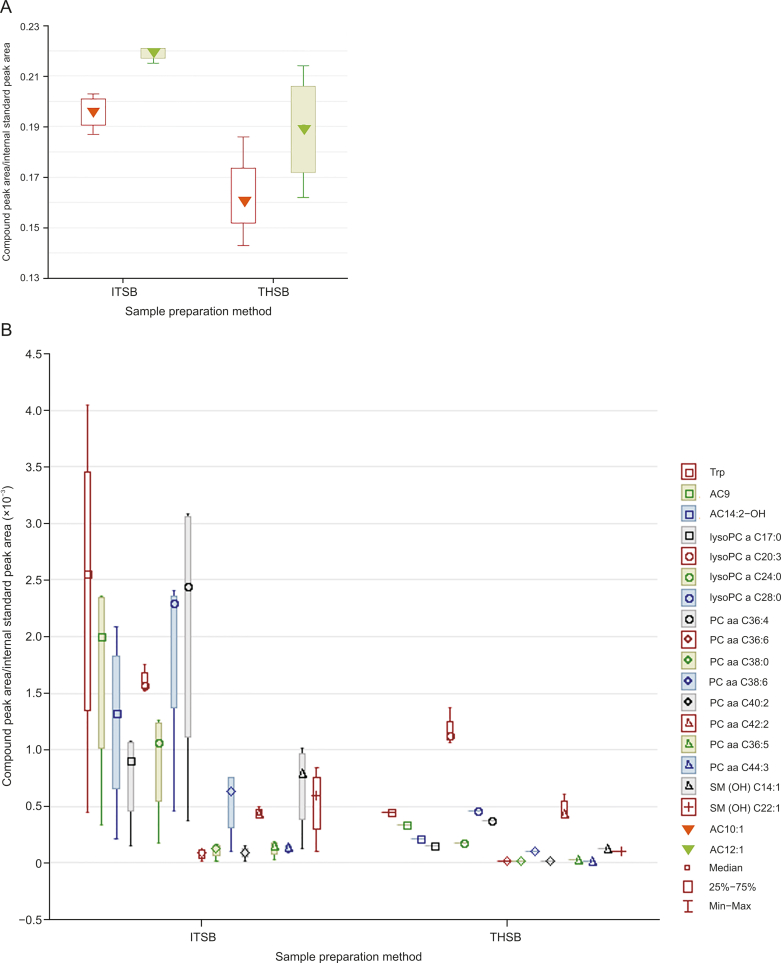
Compounds detected in lower abundance from homogenized tissue (THSB) than intact tissue (ITSB) by solid-phase microextraction (SPME) coupled to the Biocrates kit. The y-axis in (A) is over 2 orders of magnitude larger than that in (B) and therefore shown separately. Y-axis values were preprocessed with Metaboanalyst 5.0 online software. The legend lists the compounds in a descending order corresponding to their appearance on the graph from left to right. THSB: tissue homogenization followed by SPME coupled with the Biocrates kit; ITSB: intact tissue extraction via SPME coupled with the Biocrates kit; Trp: tryptophan; AC: acylcarnitine; lysoPC a: acyl-lysophosphatidylcholine; PC aa: diacyl-phosphatidylcholine; SM: sphingomyelin.

More importantly, a clear distinction was observed between the extractions from intact tissue (ITSB) and homogenized tissue (THSB) using identical SPME methods (along the principal component 2 axis). These differences are due to the destructive nature of tissue homogenization which drastically alters the types and amounts of metabolites in a sample. This finding agreed with numerous prior studies, which have speculated that the many significant differences observed in extracted metabolites can be attributed to tissue homogenization [3,10,11]. SPME fibers typically measure no more than 250 μm in width (outer diameter), which results in minimal damage to the sampled matrix [5,6]. As such, fiber-based SPME can be counted among the novel sample preparation techniques that allow for the direct analysis of intact tissue in vivo. Moreover, SPME extracts analytes via free concentration from the area closely surrounding the fiber, which enables it to determine the spatial resolution of analytes within the organ tissue, information that is generally lost during the homogenization step [20]. Such spatial variation can be seen in Fig. 1, where the variance in ITSB was larger than the variance in THSB, and the only difference was the lack of homogenization of the sample tissue. More fibers can be employed to capture a more representative metabolome profile along with spatially resolved information. Extensive tissue-analyte binding also enables rapid extraction equilibration when using in vivo SPME, which means that quantitative data can be collected in short sampling time [21]. Fig. 2, Fig. 3 illustrate the significant differences observed for all compounds during extractions from homogenized and intact tissue samples using identical SPME methods. Notably, homogenization resulted in significant changes in the concentration of small metabolites, as well as the lipidome. Specifically, the 30 compounds released by homogenization include phosphatidylcholines (PCs) with aliphatic chains containing 30 to 42 carbons, sphingomyelins (SM) with chains containing 16 to 24 carbons, methionine, and serotonin (Fig. 2). In contrast, five lysophosphatidylcholines (lysoPC) with chains containing 17 to 28 carbons, PCs with chains containing 36 to 44 carbons, two acylcarnitines (AC), two SMs, and tryptophan (in total, 17 compounds) decreased or were lost during the tissue disruption protocol (Fig. 3).

To the best of our knowledge, there exist few studies that have investigated the effect of homogenization on lysoPC levels in the tissue, which is likely due to the lack of an approach that allows these compounds to be monitored without destructive sampling/extraction techniques. Given that SPME extracts from the free concentration of analytes [22], the loss of lysoPC upon tissue homogenization can be potentially explained by several mechanisms. First, lysophosphatidylcholine acyltransferase (LPCAT), which rapidly converts lysoPC to PC as part of the Lands cycle, may be released during the homogenization step. LPCAT is an intracellular enzyme that can normally access lysoPCs only when it is transported into the cell [23,24]. The loss of lysoPC may also be due to the upregulation of autotaxin, an enzyme that facilitates the hydrolytic conversion of lysoPC to lysophosphatidic acid (LPA). LPA transmits signals calling for cell proliferation and survival, the need for which can be induced by the homogenization step [23]. Similarly, tissue and cellular disruptions can produce a greater free concentration of PCs (especially long-chain PCs) in the lung homogenate. These free long-chain PCs may be created by LPCAT activity discussed above or released from cell membranes during the homogenization process [25]. Further studies are required to confirm these hypotheses. Nevertheless, this study demonstrated a clear metabolome change due to homogenization during sample preparation.

**Fig. 4:**
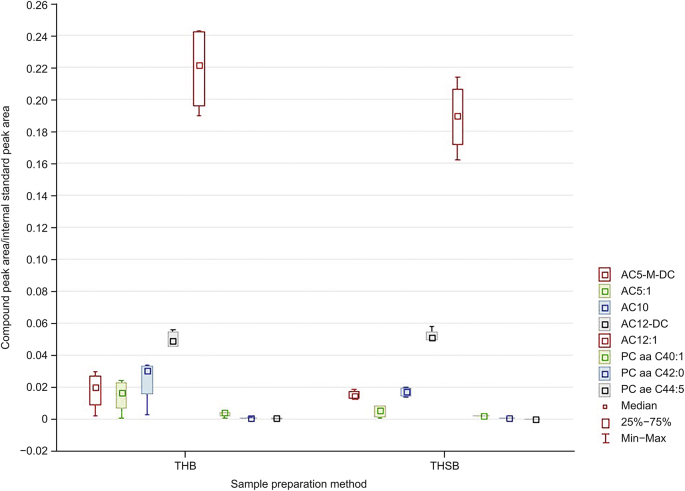
Compounds with similar amounts detected by solid-phase microextraction (SPME) (THSB) and solid-liquid extraction (THB) from homogenized tissue. Y-axis values were preprocessed with Metaboanalyst 5.0 online software. The legend lists the compounds in descending order corresponding to their appearance on the graph from left to right. THB: tissue homogenization followed by solid-liquid extraction (SLE) coupled with the Biocrates kit; THSB: tissue homogenization followed by SPME coupled to the Biocrates kit; AC: acylcarnitine; DC: dicarboxyl; PC: phosphatidylcholine. PC aa: diacyl-PC; PC ae: acyl-alkyl-PC.

As shown in Fig. 4, the compounds with similar recoveries between THB and THSB mostly comprised ACs and a few very long-chain PCs. The similarity in the amounts of long-chain PCs is not surprising, for they are very low in natural abundance; however, the similarity in these ACs was likely due to SPME's enrichment effect on the extracted analytes when using small desorption volumes [22]. Furthermore, the C8 and the anionic sulfonate functional groups in the mixed-mode extraction phase chemistry also facilitate the extraction of the carnitine head group [26]. Acylcarnitines are intermediate lipid molecules that play a key role in fatty acid transportation in the mitochondria as part of the fatty acid oxidation (FAO) cycle. These molecules are abundant in all biological fluids, especially the liquid portion of blood, and are often screened for the detection of long-chain FAO disorders [27,28]. As such, it is unsurprising that comparable amounts of many acylcarnitine compounds were extracted via SLE and SPME. SPME's ability to extract carnitines was also demonstrated in a previous study wherein SPME fibers were applied to samples from primary brain tumor tissue. Using SPME, the authors also revealed that higher-grade brain tumors contained higher carnitine levels, and that a relationship existed between carnitine levels and isocitrate dehydrogenase mutation status [11].

**Fig. 5:**
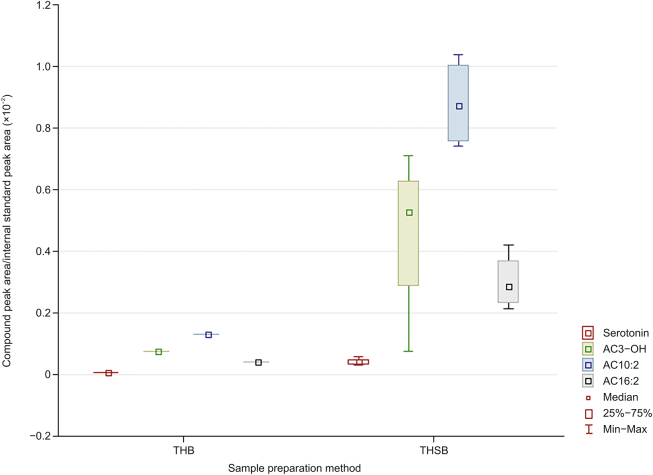
Compounds better detected by solid-phase microextraction (SPME) (THSB) than solid-liquid extraction (SLE). Y-axis values were pre-processed with Metaboanalyst 5.0 online software. THB: tissue homogenization followed by SLE coupled with the Biocrates kit; THSB: tissue homogenization followed by SPME coupled to the Biocrates kit; AC: acylcarnitine.

Despite SPME's nonexhaustive nature, the wide coverage of endogenous analytes was still expected due to its sample cleanup and concentrating effects [5,22]. SPME analysis can be quantitative despite extracting smaller quantities relative to conventional exhaustive techniques, as chromatographic baseline noise is greatly reduced/near-zero in this technique. Furthermore, the small amounts extracted in SPME also allow for wide analyte coverage in the extraction phase. Sample cleanup was achieved with the biocompatible polyacrylonitrile-based binder in the sorbent coating, which prevented macromolecules from interacting with the extraction phase. This binder also enables metabolism quenching, leading to a much cleaner background than that of the traditional SLE method [22]. In terms of analyte coverage, Birjandi et al. [29] demonstrated the advantage of using SPME for the nonexhaustive extraction of lipids from human hepatocellular carcinoma cells compared to the more conventional SLE method. While SLE extracted a great abundance of phospholipids, SPME provided a more balanced recovery, which greatly reduced matrix effects during LC-MS analysis, thus enabling the detection of a more diverse range of lipid classes. In the present study, the differences between the THSB and THB sample preparation methods were attributable to SPME's nonexhaustive nature, especially in the preequilibrium regime of kinetic extraction (THSB). On the other hand, while the conventional method (THB) could extract an abundance of compounds (Table S1), similar analyte coverage was obtained using SPME (THSB), with quantitative signals detected for 102 and 90 compounds for THB and THSB, respectively. Additionally, no significant differences (*P* > 0.05) between THB and THSB were observed for eight compounds (Fig. 4), and four compounds were only reliably detected via the THSB method (*P* < 0.05). Notably, these compounds were also detected in THB, but with extremely poor reproducibility (RSD > 30%). Therefore, they were excluded from the results (Fig. 5).

SPME is likely to provide more representative metabolomic information by bypassing the disruptive homogenization step. Moreover, various strategies can be employed to further increase the coverage and detection limits of SPME, such as using multiple fibers and different coating chemistries. For example, Bogusiewicz et al. [11] demonstrated that a single device comprising multiple fibers with the same coating chemistries can be implemented to perform untargeted metabolomics and lipidomics during intraoperative in vivo sampling. While the authors took advantage of one type of coating chemistry, different coating chemistries can be employed to expand analyte coverage. Desorbing multiple fibers into a single aliquot of desorption solution is also a valid quantitative approach that can drastically boost analyte recovery due to the linear relationship between the amount of analytes extracted and the number of fibers used [30].

## 4 Conclusion

The data reported in this study demonstrate that combining SPME with a well-known metabolomic profiling kit is a desirable approach for metabolome studies of undisturbed samples. This study's key findings include the discovery of drastic differences in the metabolomic profiles of intact and homogenized tissue, which have been the subject of much speculation in literature. While exhaustive extraction methods, such as SLE, can recover large quantities of metabolites from homogenized tissue biopsies, these methods often provide metabolomic profiles unrepresentative of living tissues under physiological conditions. In contrast, SPME's ability to provide noninvasive and nonexhaustive analyte recovery makes it a valuable tool for applications that involve in vivo metabolomics and do not require extensive or disruptive sample preparation steps.

## CRediT author statement

**Runshan Will Jiang**: Methodology, Validation, Formal analysis, Investigation, Data curation, Writing - Original draft preparation, Reviewing and Editing, Visualization; **Karol Jaroch**: Conceptualization, Methodology, Validation, Formal analysis, Investigation, Data curation, Writing - Reviewing and Editing, Visualization; **Janusz Pawliszyn**: Resources, Writing - Reviewing and Editing, Supervision, Funding acquisition.

## Declaration of competing interest

The authors declare that there are no conflicts of interest (including Biocrates Life Sciences).
