# Supplementary material for: Solid-phase microextraction of endogenous metabolites from intact tissue validated using a Biocrates standard reference method kit
Source: J Pharm Anal. 2022 Oct 8;13(1):55–62. doi: 10.1016/j.jpha.2022.09.002 (PMC9937786; doi:10.1016/j.jpha.2022.09.002)
Supplement: Supplementary file 1 — Multimedia component 1 [file mmc1.docx]

**Supplementary data**

**Solid-phase microextraction of endogenous metabolites from intact tissue validated using a Biocrates standard reference method kit.**

**Table S1.** Mass spectrometry (MS) analyte-to-internal standard ratios of compounds detected by each sample preparation method.

| **#** | **Compound** | **Compound class** | **Detection method** | **P180 kit (THB)** | **SPME p180 kit (THSB)** | **SPME p180 kit (ITSB)** |
| --- | --- | --- | --- | --- | --- | --- |
| 1 | Met | Amino acids | LC-MS | 1.5 (1×10^−1^) | 0.0009 (1×10^−1^) | ND ^*^ |
| 2 | Phe |  |  | 3.5 (3×10^−1^) | ND | 0.011 (3×10^−3^) |
| 3 | Trp |  |  | 0.54 (4×10^−2^) | ND | 0.0030 (9×10^−4^) |
| 1 | Serotonin | Biogenic amines |  | ND | 0.0004 (1×10^−4^) | ND |
| 2 | Taurine |  |  | 13.1 (1×10^−1^) | 0.032 (8×10^−3^) | ND |
| 1 | AC3-OH | Acylcarnitines | FIA | ND | 0.006 (1×10^−3^) | 0.005 (1×10^−3^) |
| 2 | AC5-M-DC |  |  | 0.023 (7×10^−3^) | 0.015 (3×10^−3^) | 0.022 (7×10^−3^) |
| 3 | AC5:1 |  |  | 0.019 (6×10^−3^) | 0.008 (1×10^−3^) | 0.008 (1×10^−3^) |
| 4 | AC8 |  |  | 0.040 (3×10^−3^) | 0.028 (3×10^−3^) | 0.026 (1×10^−3^) |
| 5 | AC9 |  |  | 0.008 (1×10^−3^) | 0.0021 (4×10^−4^) | ND |
| 6 | AC10 |  |  | 0.031 (3×10^−3^) | 0.018 (2×10^−3^) | 0.0168 (9×10^−4^) |
| 7 | AC10:1 |  |  | 0.19 (1×10^−2^) | 0.17 (1×10^−2^) | 0.199 (5×10^−3^) |
| 8 | AC10:2 |  |  | ND | 0.009 (1×10^−3^) | 0.0077 (1×10^−4^) |
| 9 | AC12 |  |  | 0.23 (2×10^−2^) | 0.013 (2×10^−3^) | 0.0124 (1×10^−4^) |
| 10 | AC12-DC |  |  | 0.050 (5×10^−3^) | 0.052 (4×10^−3^) | 0.056 (3×10^−3^) |
| 11 | AC12:1 |  |  | 0.22 (3×10^−2^) | 0.19 (3×10^−2^) | 0.219 (3×10^−3^) |
| 12 | AC14 |  |  | 0.014 (×10^−3^) | 0.0050 (7×10^−4^) | 0.0040 (5×10^−4^) |
| 13 | AC14:2-OH |  |  | 0.0025 (3×10^−4^) | ND | 0.0016 (5×10^−4^) |
| 14 | AC16:1 |  |  | 0.013 (3×10^−3^) | 0.003 (6×10^−4^) | 0.0026 (5×10^−4^) |
| 15 | AC16:2 |  |  | ND | 0.0030 (9×10^−4^) | 0.0034 (1×10^−3^) |
| 16 | AC16:2-OH |  |  | 0.006 (1×10^−3^) | ND | 0.0029 (5×10^−4^) |
| 1 | lysoPC C14:0 | Glycero-phospholipids |  | 0.021 (3×10^−3^) | 0.0029 (2×10^−4^) | 0.0032 (4×10^−4^) |
| 2 | lysoPC C16:0 |  |  | 0.31 (4×10^−2^) | 0.0051 (8×10^−4^) | 0.0104 (7×10^−4^) |
| 3 | lysoPC C16:1 |  |  | 0.031 (2×10^−3^) | 0.0019 (3×10^−4^) | 0.0023 (3×10^−4^) |
| 4 | lysoPC C17:0 |  |  | 0.025 (2×10^−3^) | ND | 0.0010 (2×10^−4^) |
| 5 | lysoPC C18:0 |  |  | 0.10 (1×10^−2^) | 0.0029 (3×10^−4^) | 0.0041 (3×10^−4^) |
| 6 | lysoPC C18:1 |  |  | 0.083 (9×10^−3^) | 0.0017 (2×10^−4^) | 0.0036 (2×10^−4^) |
| 7 | lysoPC C18:2 |  |  | 0.023 (3×10^−3^) | ND | 0.0017 (2×10^−4^) |
| 8 | lysoPC C20:3 |  |  | 0.0046 (3×10^−4^) | 0.0012 (1×10^−4^) | 0.0016 (1×10^−4^) |
| 9 | lysoPC C20:4 |  |  | 0.017 (2×10^−3^) | 0.0009 (1×10^−4^) | 0.0014 (1×10^−4^) |
| 10 | lysoPC C24:0 |  |  | 0.008 (1×10^−3^) | ND | 0.0011 (2×10^−4^) |
| 11 | lysoPC C26:0 |  |  | 0.023 (3×10^−3^) | 0.0014 (3×10^−4^) | 0.0030 (7×10^−4^) |
| 12 | lysoPC C26:1 |  |  | 0.006 (1×10^−3^) | 0.0011 (3×10^−4^) | 0.0019 (3×10^−4^) |
| 13 | lysoPC C28:0 |  |  | 0.022 (2×10^−3^) | ND | 0.0023 (1×10^−4^) |
| 14 | lysoPC C28:1 |  |  | 0.012 (1×10^−3^) | 0.0012 (3×1^0−4^) | ND |
| 1 | PC aa C24:0 |  |  | 0.0058 (7×10^−4^) | 0.0008 (2×10^−4^) | 0.013 (2×10^−3^) |
| 2 | PC aa C26:0 |  |  | 0.029 (5×10^−3^) | 0.008 (2×10^−3^) | 0.013 (2×10^−3^) |
| 3 | PC aa C28:1 |  |  | 0.012 (1×10^−3^) | 0.0013 (3×10^−4^) | 0.0018 (4×10^−4^) |
| 4 | PC aa C30:0 |  |  | 1.12 (8×10^−2^) | 0.005 (1×10^−3^) | ND |
| 5 | PC aa C32:0 |  |  | 2.4 (2×10^−1^) | 0.006 (1×10^−3^) | ND |
| 6 | PC aa C32:1 |  |  | 0.99 (7×10^−2^) | 0.0031 (6×10^−4^) | ND |
| 7 | PC aa C32:2 |  |  | 0.050 (4×10^−3^) | 0.0005 (1×10^−4^) | 0.0007 (2×10^−4^) |
| 8 | PC aa C32:3 |  |  | 0.0040 (4×10^−4^) | 0.0001 (<1×10^−4^) | ND |
| 9 | PC aa C34:1 |  |  | 0.0040 (4×10^−4^) | 0.004 (1×10^−3^) | ND |
| 10 | PC aa C34:2 |  |  | 1.25 (9×10^−2^) | 0.0024 (6×10^−4^) | ND |
| 11 | PC aa C34:3 |  |  | 0.051 (2×10^−3^) | 0.0003 (<1×10^−4^) | 0.0006 (1×10^−4^) |
| 12 | PC aa C34:4 |  |  | 0.0103 (7×10^−4^) | 0.0002 (<1×10^−4^) | 0.0002 (<1×10^−4^) |
| 13 | PC aa C36:0 |  |  | 0.0026 (6×10^−4^) | 0.0006 (<1×10^−4^) | 0.0006 (1×10^−4^) |
| 14 | PC aa C36:2 |  |  | 0.34 (3×10^−2^) | 0.0015 (5×10^−4^) | 0.0023 (6×10^−4^) |
| 15 | PC aa C36:3 |  |  | 0.17 (1×10^−2^) | 0.0007 (1×10^−4^) | ND |
| 16 | PC aa C36:4 |  |  | 0.35 (2×10^−2^) | ND | 0.0027 (7×10^−4^) |
| 17 | PC aa C36:5 |  |  | 0.039 (2×10^−3^) | 0.0004 (1×10^−4^) | ND |
| 18 | PC aa C36:6 |  |  | 0.0038 (6×10^−4^) | ND | 0.0001 (<1×10^−4^) |
| 19 | PC aa C38:0 |  |  | 0.0054 (3×10^−4^) | ND | 0.0001 (<1×10^−4^) |
| 20 | PC aa C38:3 |  |  | 0.052 (4×10^−4^) | 0.0003 (<1×10^−4^) | ND |
| 21 | PC aa C38:4 |  |  | 0.18 (1×10^−2^) | 0.0007 (2×10^−4^) | ND |
| 22 | PC aa C38:5 |  |  | 0.122 (8×10^−3^) | 0.0007 (2×10^−4^) | ND |
| 23 | PC aa C38:6 |  |  | 0.029 (2×10^−3^) | ND | 0.0007 (2×10^−4^) |
| 24 | PC aa C40:1 |  |  | 0.0042 (2×10^−4^) | 0.0024 (<1×10^−4^) | 0.0023 (<1×10^−4^) |
| 25 | PC aa C40:2 |  |  | 0.0023 (3×10^−4^) | ND | 0.0001 (<1×10^−4^) |
| 26 | PC aa C40:4 |  |  | 0.0133 (5×10^−4^) | 0.0002 (1×10^−4^) | 0.0002 (1×10^−4^) |
| 27 | PC aa C40:5 |  |  | 0.023 (1×10^−3^) | 0.0001 (<1×10^−4^) | ND |
| 28 | PC aa C40:6 |  |  | 0.018 (1×10^−3^) | 0.0009 (2×10^−4^) | 0.0010 (2×10^−4^) |
| 29 | PC aa C42:0 |  |  | 0.0010 (2×10^−4^) | 0.0007 (2×10^−4^) | 0.0006 (<1×10^−4^) |
| 30 | PC aa C42:2 |  |  | 0.018 (1×10^−4^) | 0.0005 (1×10^−4^) | 0.0005 (<1×10^−4^) |
| 31 | PC aa C42:6 |  |  | 0.0021 (1×10^−4^) | 0.0005 (<1×10^−4^) | 0.0006 (<1×10^−4^) |
|  |  |  |  |  |  |  |
| 1 | PC ae C30:0 |  |  | 0.12 (1×10^−2^) | 0.0014 (1×10^−4^) | 0.0022 (6×10^−4^) |
| 2 | PC ae C30:2 |  |  | 0.0023 (6×10^−4^) | 0.0002 (<1×10^−4^) | 0.0003 (<1×10^−4^) |
| 3 | PC ae C32:1 |  |  | 0.091 (9×10^−3^) | 0.0005 (1×10^−4^) | ND |
| 4 | PC ae C34:1 |  |  | 0.27 (2×10^−32^) | 0.0009 (2×10^−4^) | 0.0025 (7×10^−4^) |
| 5 | PC ae C34:2 |  |  | 0.04 (3×10^−3^) | 0.0003 (<1×10^−4^) | ND |
| 6 | PC ae C34:3 |  |  | 0.0071 (9×10^−4^) | 0.0002 (<1×10^−4^) | 0.0003 (<1×10^−4^) |
| 7 | PC ae C36:0 |  |  | 0.015 (2×10^−3^) | 0.0005 (<1×10^−4^) | 0.0005 (1×10^−4^) |
| 8 | PC ae C36:2 |  |  | 0.065 (5×10^−3^) | 0.0004 (<1×10^−4^) | 0.0007 (2×10^−4^) |
| 9 | PC ae C36:3 |  |  | 0.0171 (5×10^−4^) | 0.0001 (<1×10^−4^) | ND |
| 10 | PC ae C36:4 |  |  | 0.028 (2×10^−3^) | 0.0001 (<1×10^−4^) | ND |
| 11 | PC ae C36:5 |  |  | 0.018 (1×10^−3^) | ND | 0.0002 (<1×10^−4^) |
| 12 | PC ae C38:0 |  |  | 0.0055 (1×10^−4^) | 0.0012 (<1×10^−4^) | 0.0013 (1×10^−4^) |
| 13 | PC ae C38:1 |  |  | 0.0074 (7×10^−4^) | 0.0003 (<1×10^−4^) | 0.0004 (1×10^−4^) |
| 14 | PC ae C38:3 |  |  | 0.0134 (9×10^−4^) | 0.0002 (<1×10^−4^) | 0.0001 (<1×10^−4^) |
| 15 | PC ae C38:4 |  |  | 0.038 (2×10^−3^) | 0.0007 (1×10^−4^) | ND |
| 16 | PC ae C38:5 |  |  | 0.032 (2×10^−3^) | 0.0002 (<1×10^−4^) | 0.0004 (<1×10^−^4) |
| 17 | PC ae C38:6 |  |  | 0.0090 (6×10^−4^) | 1×10^−4^ (<1×10^−4^) | ND |
| 18 | PC ae C40:1 |  |  | 0.004 (1×10^−4^) | 0.002 (<1×10^−4^) | ND |
| 19 | PC ae C40:4 |  |  | 0.0083 (7×10^−4^) | 0.0003 (<1×10^−4^) | 0.0003 (<1×10^−4^) |
| 20 | PC ae C40:5 |  |  | 0.009 (1×10^−3^) | 0.0001 (<1×10^−4^) | 0.0001 (<1×10^−4^) |
| 21 | PC ae C40:6 |  |  | 0.0067 (6×10^−4^) | 0.0001 (<1×10^−4^) | 0.0001 (<1×10^−4^) |
| 22 | PC ae C42:0 |  |  | 0.0035 (3×10^−4^) | 0.0017 (<1×10^−4^) | 0.0018 (<1×10^−4^) |
| 23 | PC ae C42:1 |  |  | 0.0029 (6×10^−4^) | 0.0002 (<1×10^−4^) | 0.0003 (<1×10^−4^) |
| 24 | PC ae C42:2 |  |  | 0.0024 (5×10^−4^) | 0.0001 (<1×10^−4^) | ND |
| 25 | PC ae C42:5 |  |  | 0.0046 (3×10^−4^) | 0.0027 (1×10^−4^) | 0.0028 (1×10^−4^) |
| 26 | PC ae C44:3 |  |  | 0.0004 (<1×10^−4^) | 0.0027 (1×10^−4^) | 0.0028 (1×10^−4^) |
| 27 | PC ae C44:4 |  |  | 0.0006 (3×10^−4^) | 0.0002 (<1×10^−4^) | 0.0001 (<1×10^−4^) |
| 28 | PC ae C44:5 |  |  | 0.0007 (1×10^−4^) | 0.0003 (<1×10^−4^) | 0.0003 (<1×10^−4^) |
| 29 | PC ae C44:6 |  |  | 0.0011 (<1×10^−4^) | 0.0005 (1×10^−4^) | 0.0005 (>×10^−4^) |
| 1 | SM(OH)C14:1 | Sphingo-myelins |  | 0.076 (6×10^−3^) | ND | 0.0009 (2×10^−4^) |
| 2 | SM(OH)C16:1 |  |  | 0.115 (9×10^−3^) | 0.0006 (1×10^−4^) | ND |
| 3 | SM(OH)C22:1 |  |  | 0.20 (2×10^−2^) | ND | 0.0007 (2×10^−4^) |
| 4 | SM(OH)C22:2 |  |  | 0.070 (6×10^−3^) | 0.0002 (<1×10^−4^) | ND |
| 5 | SM(OH)C24:1 |  |  | 0.092 (7×10^−3^) | 0.0002 (<1×10^−4^) | 0.0002 (<1×10^−4^) |
| 6 | SM C16:0 |  |  | 1.1 (1×10^−1^) | 0.006 (1×10^−3^) | ND |
| 7 | SM C16:1 |  |  | 0.029 (4×10^−3^) | 0.0003 (<1×10^−4^) | ND |
| 8 | SM C18:0 |  |  | 0.122 (7×10^−3^) | 0.0005 (1×10^−4^) | ND |
| 9 | SM C18:1 |  |  | 0.0138 (7×10^−4^) | 0.0001 (<1×10^−4^) | ND |
| 10 | SM C24:0 |  |  | 0.45 (3×10^−2^) | 0.0007 (2×10^−4^) | ND |
| 11 | SM C24:1 |  |  | 0.38 (4×10^−2^) | 0.0006 (1×10^−4^) | ND |

^*^ ND: not determined (due to signal-to-noise (S:N) ratio < 3 and/or relative standard deviation (RSD%) > 30%). THB: tissue homogenization followed by solid-liquid extraction (SLE) coupled with the Biocrates kit; THSB: tissue homogenization followed by solid-phase microextraction (SPME) coupled with the Biocrates kit; ITSB: intact tissue extraction via SPME coupled with the Biocrates kit; Met: methionine; LC: liquid chromatography; Phe: phenylalanine; Trp: tryptophan; AC: acylcarnitine; FIA: flow injection analysis; DC: dicarboxyl; lysoPC: lysophosphatidylcholine; PC: phosphatidylcholine; SM: sphingomyelin.


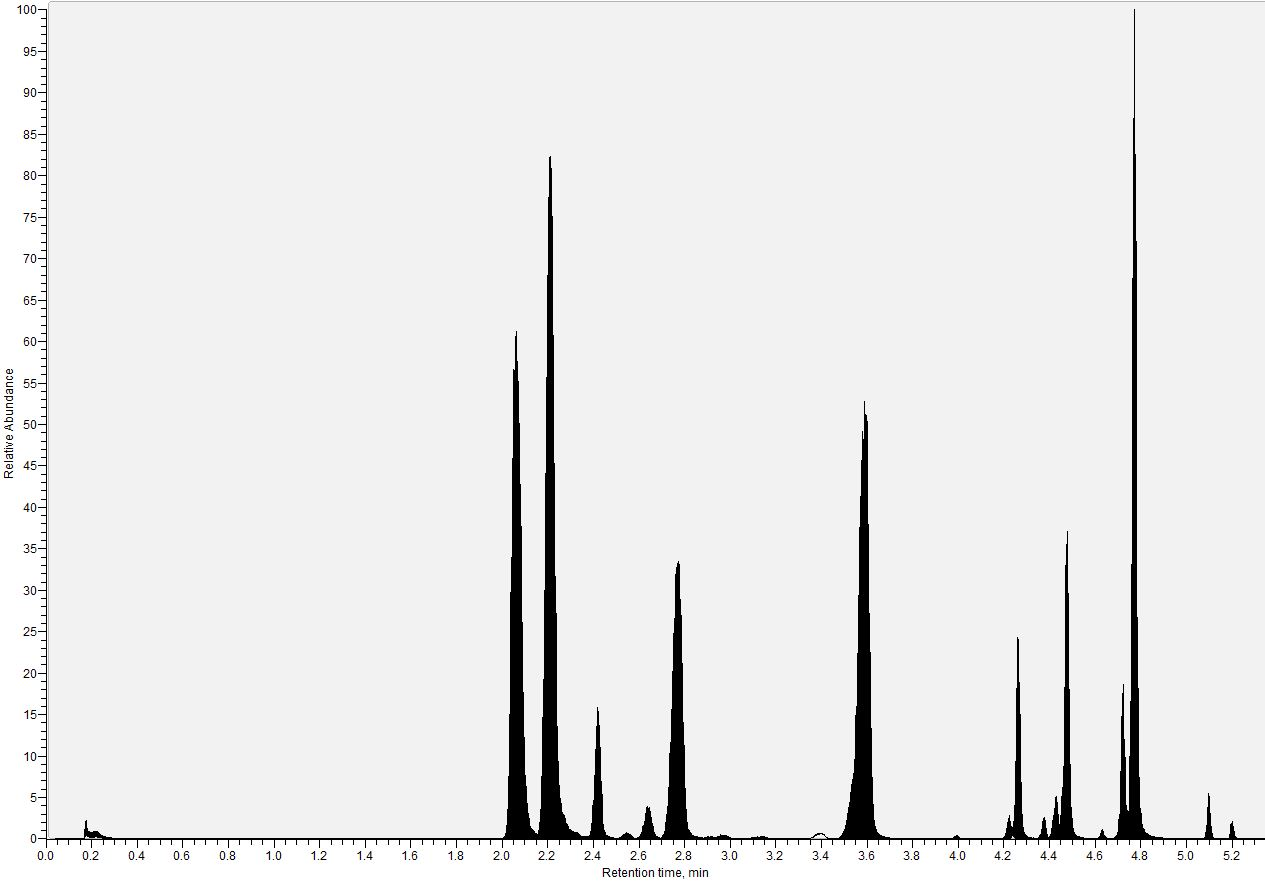


**Fig. S1.** Exemplary liquid chromatography-mass spectrometry (LC-MS) total ion current chromatogram for tissue homogenization followed by solid-liquid extraction (SLE) coupled with the Biocrates kit (THB) (intensity: 7.62×10^6^).

**
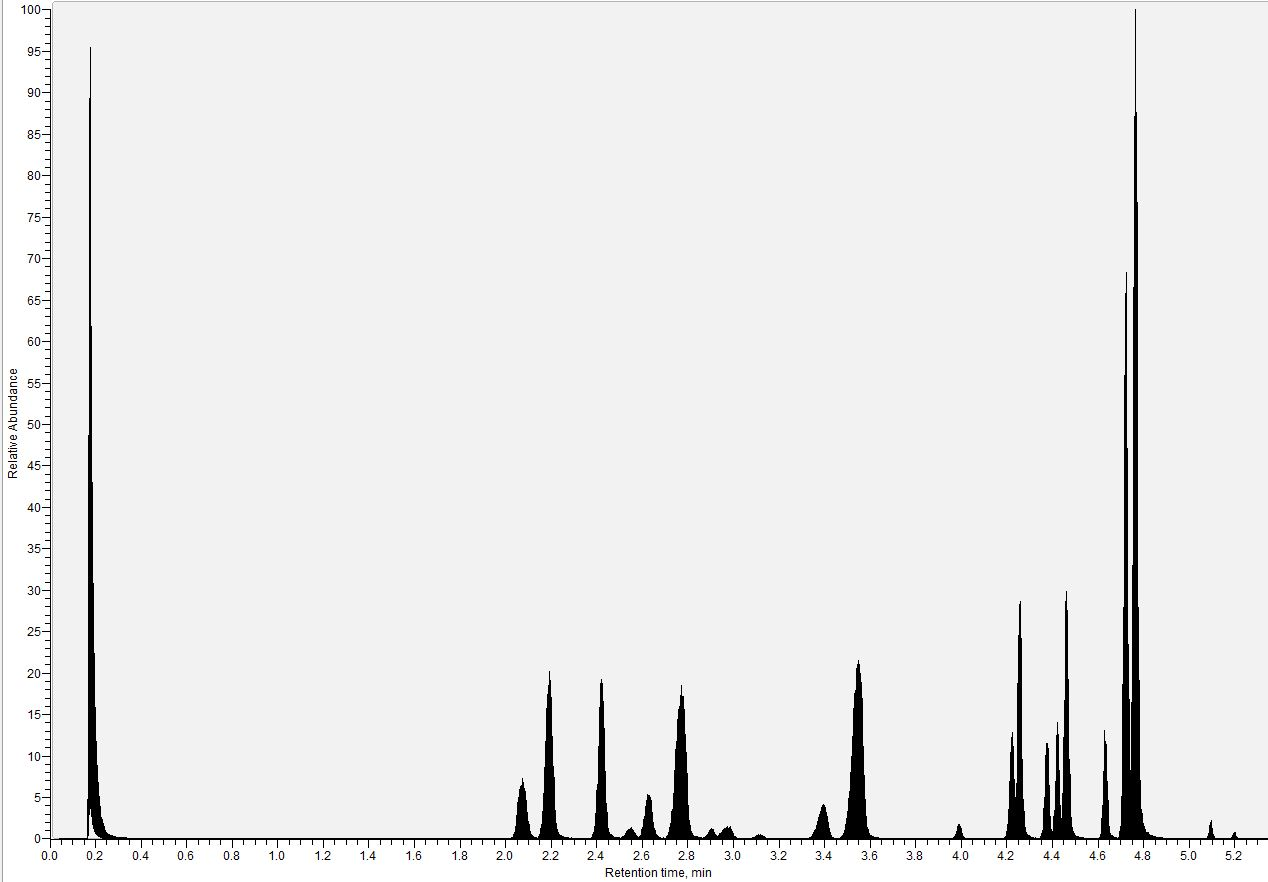
**

**Fig. S2.** Exemplary liquid chromatography-mass spectrometry (LC-MS) total ion current chromatogram for tissue homogenization followed by solid-phase microextraction (SPME) coupled with the Biocrates kit (THSB) (intensity: 3.34×10^6^).

**
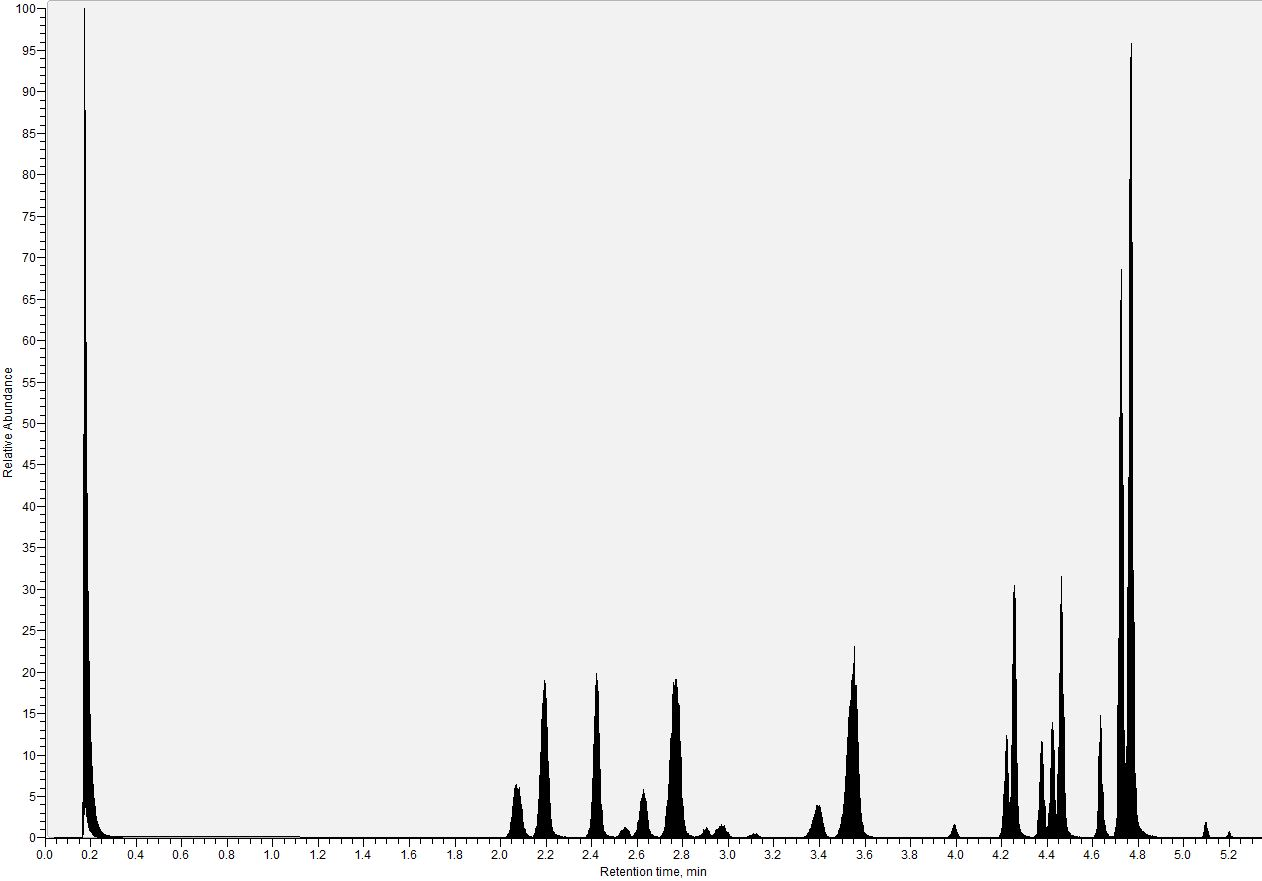
**

**Fig. S3.** Exemplary liquid chromatography-mass spectrometry (LC-MS) total ion current chromatogram for intact tissue extraction via solid-phase microextraction (SPME) coupled with the Biocrates kit (ITSB) (intensity: 3.14×10^6^).

**
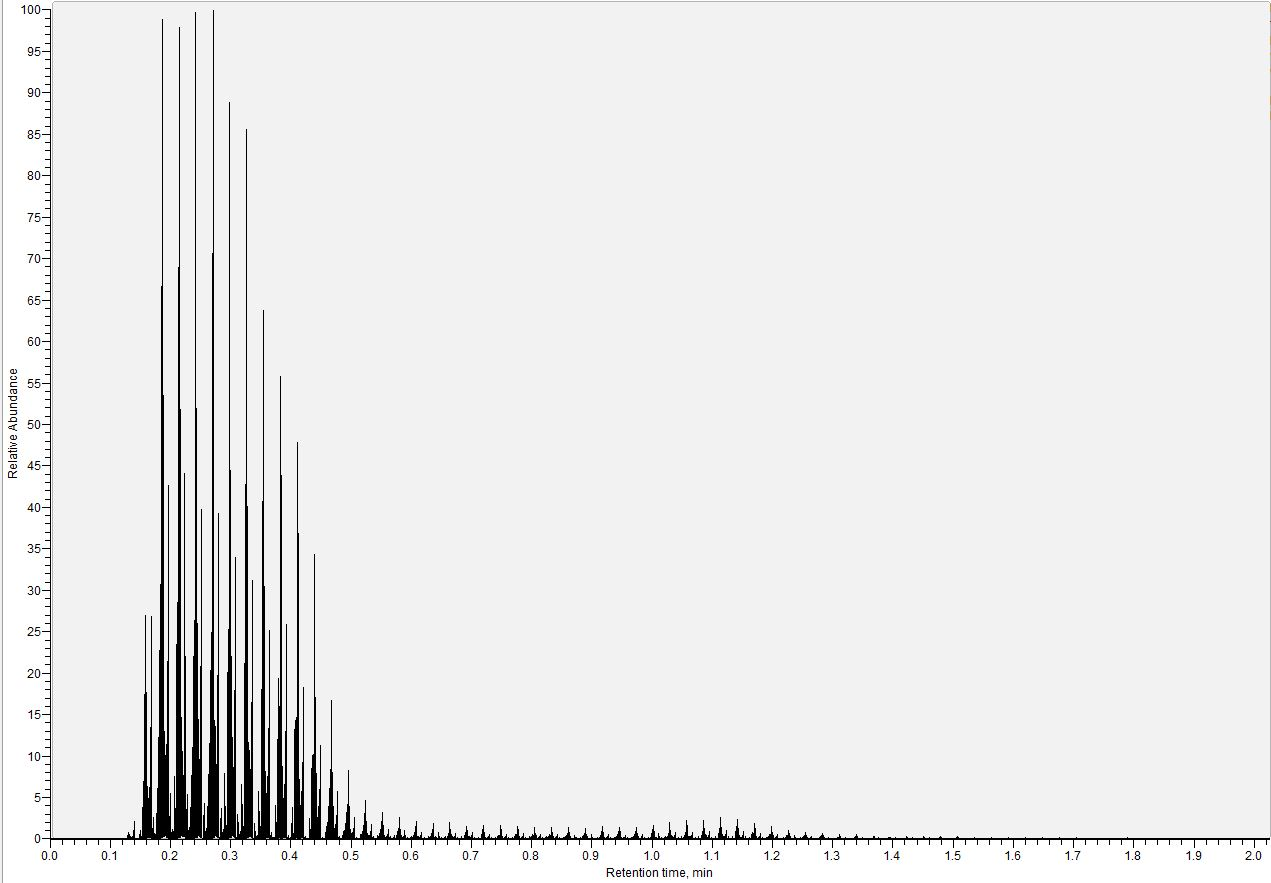
**

**Fig. S4.** Exemplary positive mode flow injection analysis (FIA) total ion current chromatogram for tissue homogenization followed by solid-liquid extraction (SLE) coupled with the Biocrates kit (THB) (intensity: 3.79×10^6^).

**
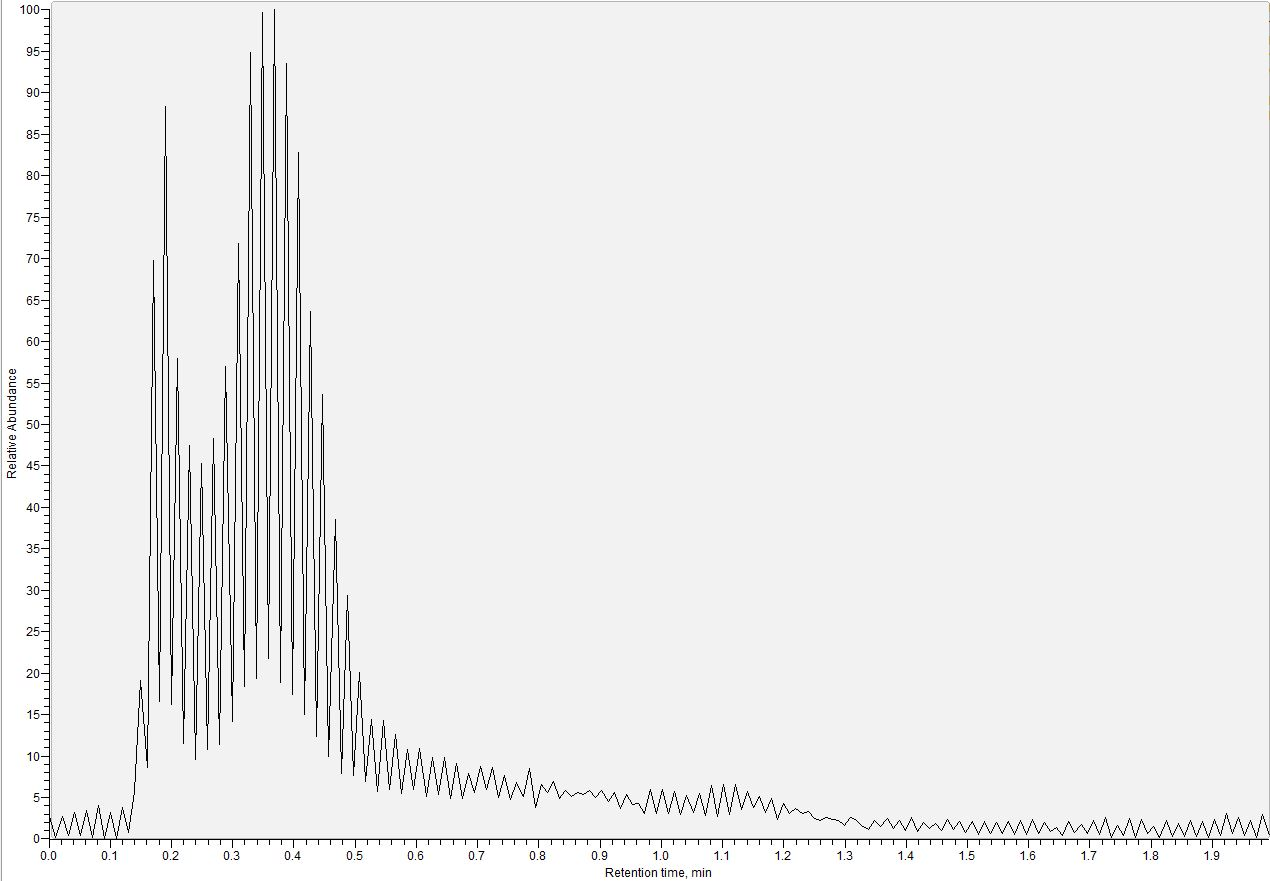
**

**Fig. S5.** Exemplary negative mode flow injection analysis (FIA) total ion current chromatogram for tissue homogenization followed by solid-liquid extraction (SLE) coupled with the Biocrates kit (THB) (intensity: 1.37×10^3^).

**
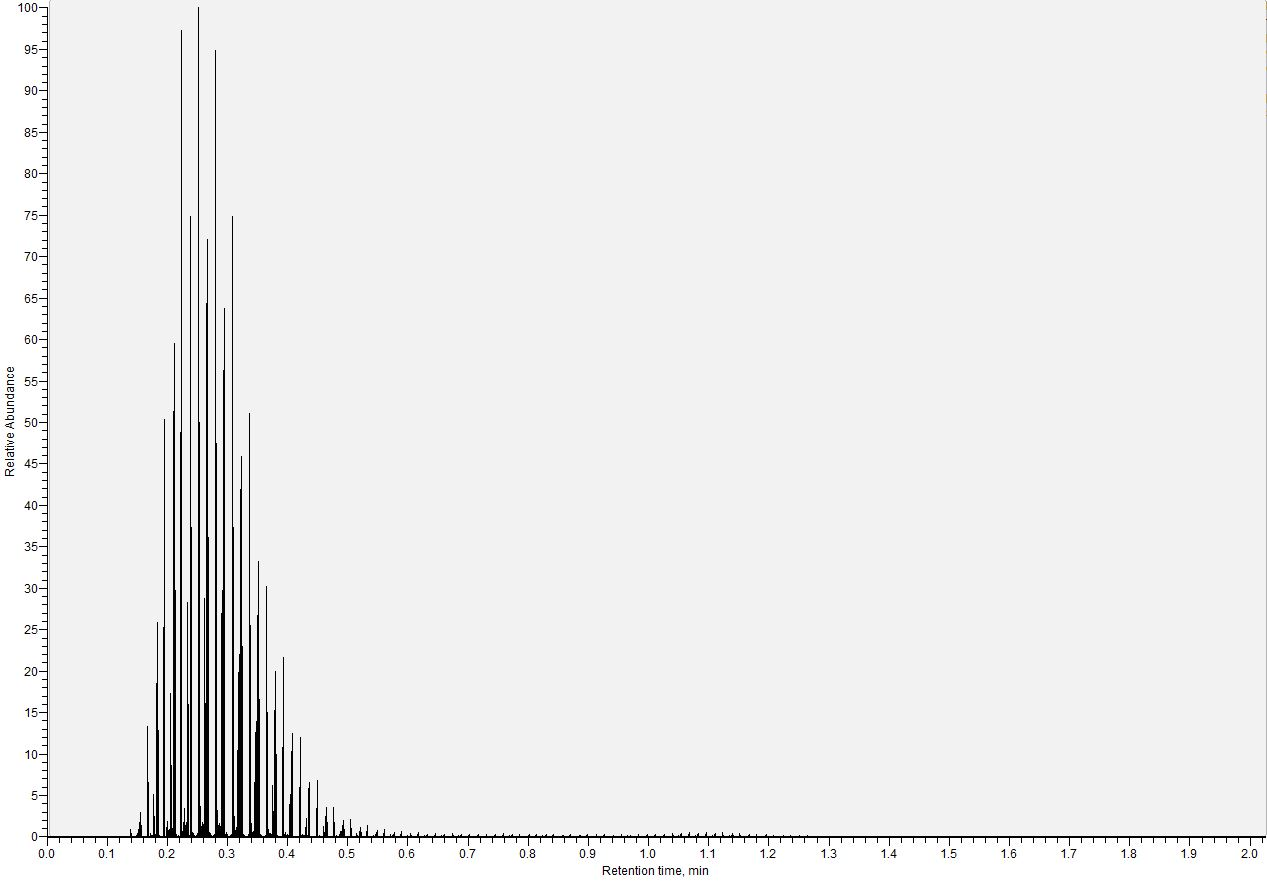
**

**Fig. S6.** Exemplary positive mode flow injection analysis (FIA) total ion current chromatogram for tissue homogenization followed by solid-phase microextraction (SPME) coupled with the Biocrates kit (THSB) (intensity: 9.14×10^6^).

**
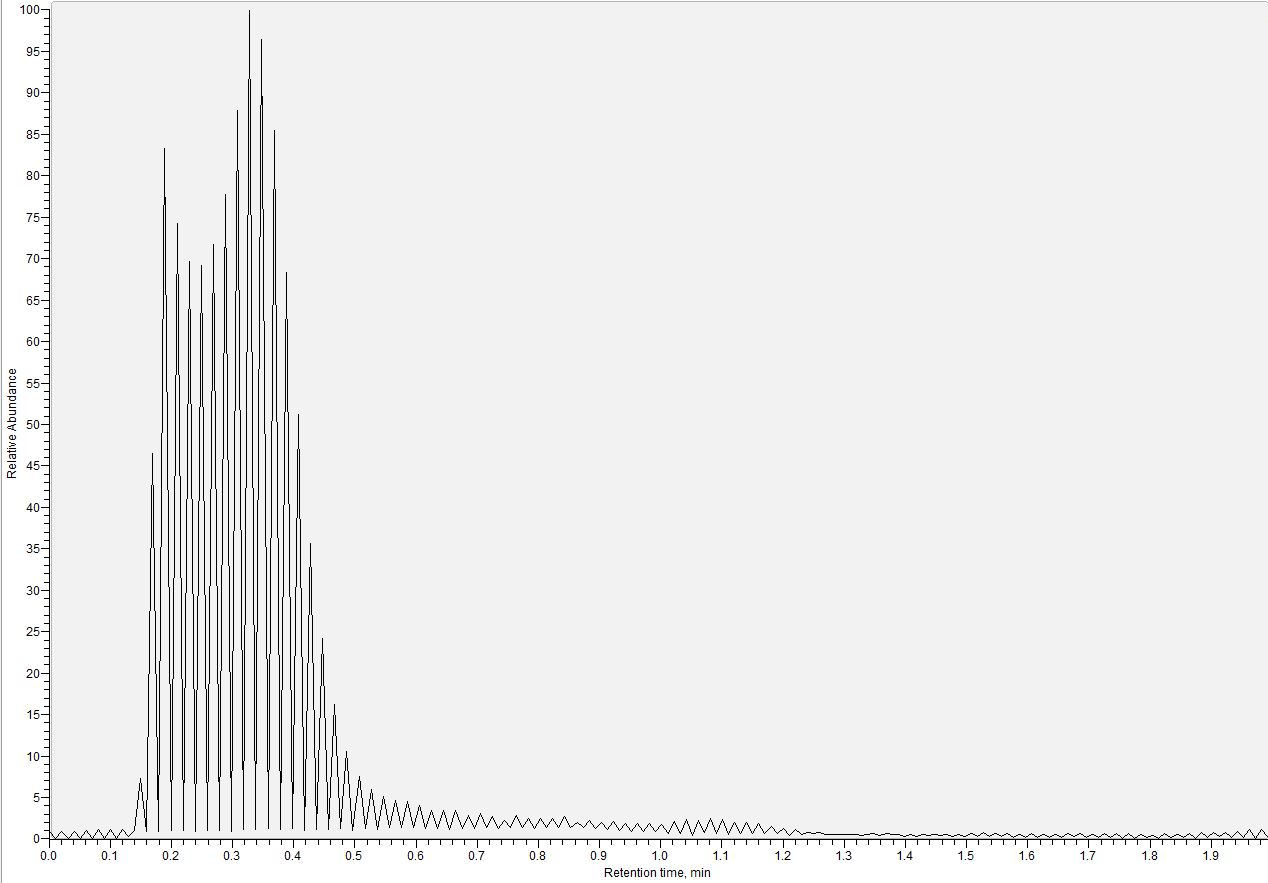
**

**Fig. S7.** Exemplary negative mode flow injection analysis (FIA) total ion current chromatogram for tissue homogenization followed by solid-phase microextraction (SPME) coupled with the Biocrates kit (THSB) (intensity: 5.18×10^3^).

**
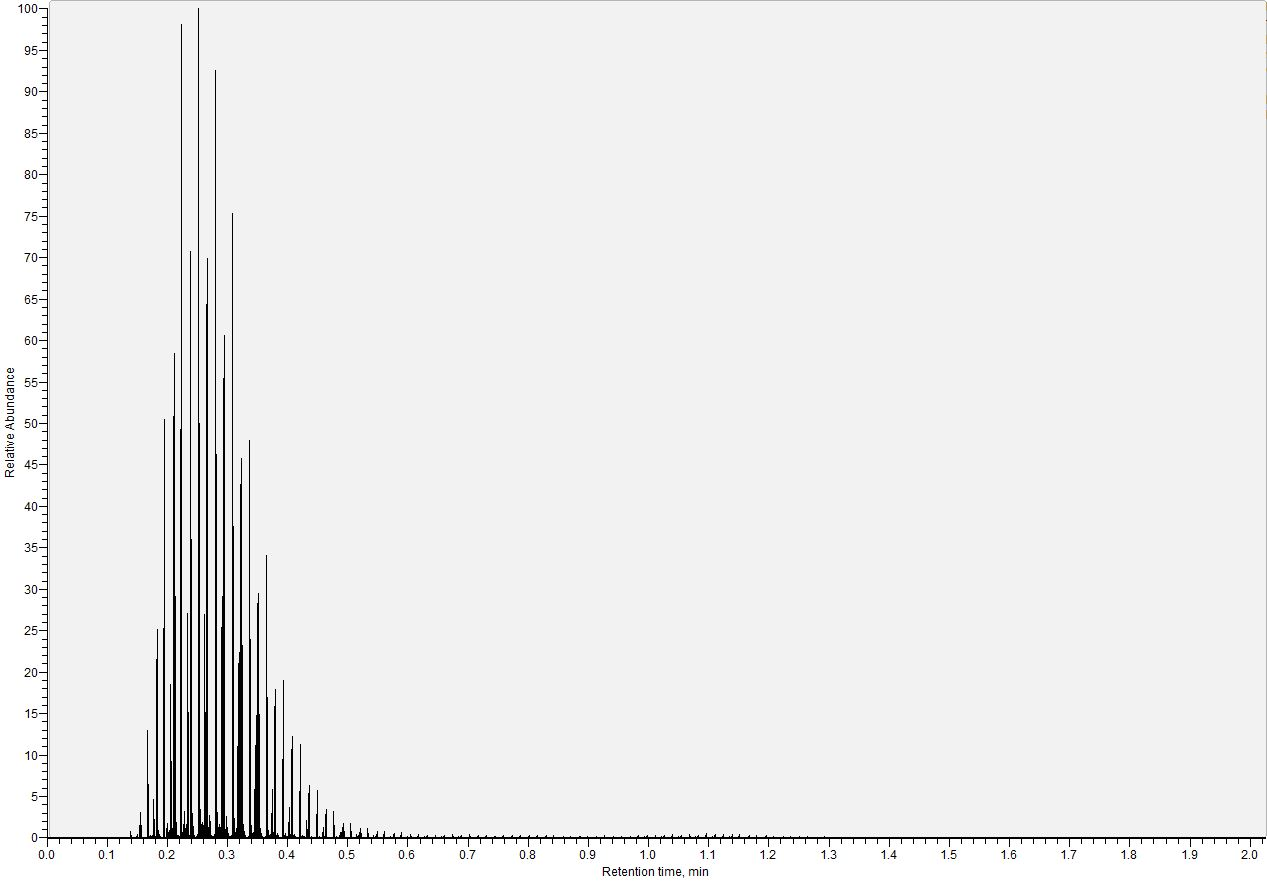
**

**Fig. S8.** Exemplary positive mode flow injection analysis (FIA) total ion current chromatogram for intact tissue extraction via solid-phase microextraction (SPME) coupled with the Biocrates kit (ITSB) (intensity: 9.12×10^6^).

**
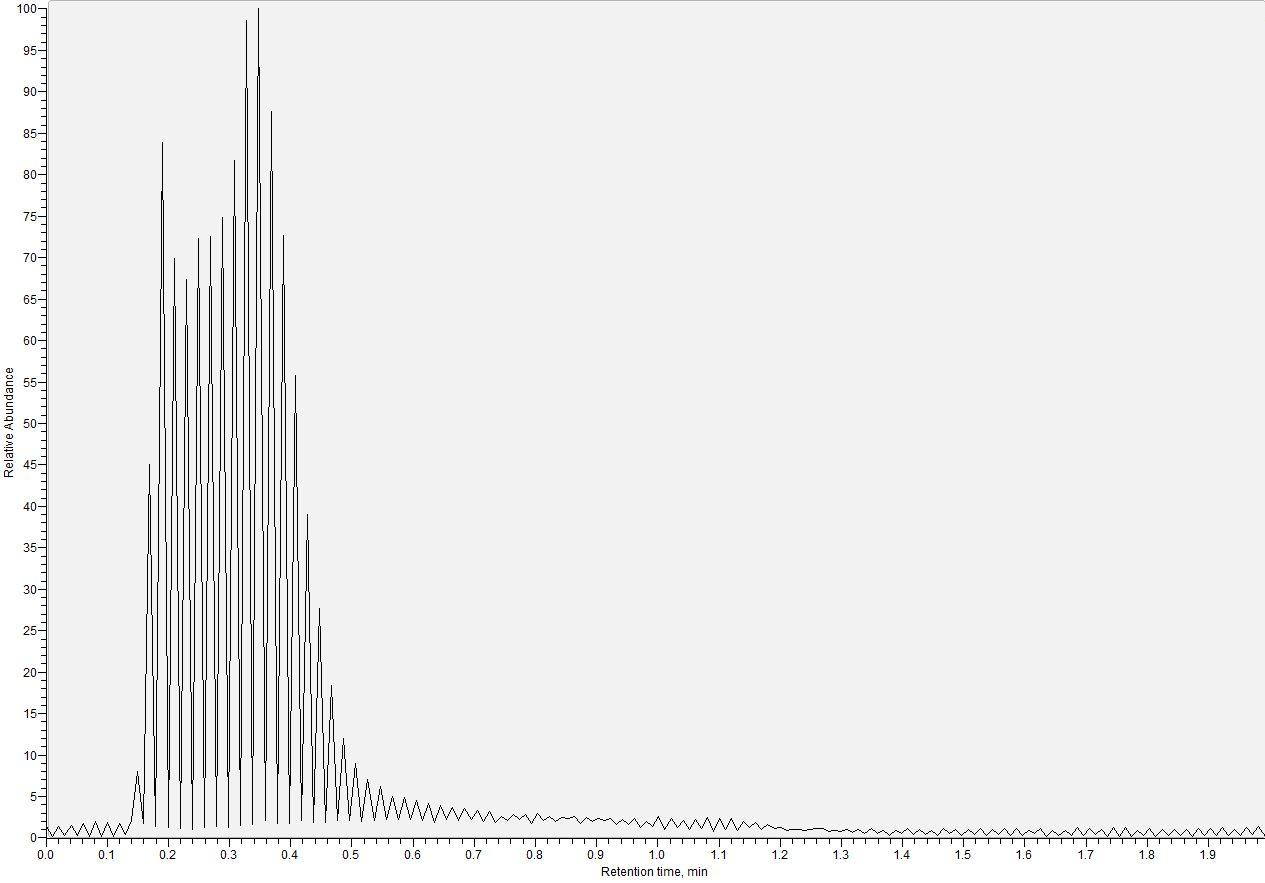
**

**Fig. S9.** Exemplary negative mode flow injection analysis (FIA) total ion current chromatogram for intact tissue extraction via solid-phase microextraction (SPME) coupled with the Biocrates kit (ITSB) (intensity: 4.68×10^3^).
